# A spiking neural network model of the midbrain superior colliculus that generates saccadic motor commands

**DOI:** 10.1007/s00422-017-0719-9

**Published:** 2017-05-20

**Authors:** Bahadir Kasap, A. John van Opstal

**Affiliations:** Department of Biophysics, Donders Institute for Brain, Cognition and Behaviour, Radboud University, HG00.800, Heyendaalseweg 135, 6525 AJ Nijmegen, The Netherlands

**Keywords:** Saccades, Superior colliculus, Motor map, Spatial–temporal transformation, Spiking neural network, Pulse generation, Nonlinearity

## Abstract

Single-unit recordings suggest that the midbrain superior colliculus (SC) acts as an optimal controller for saccadic gaze shifts. The SC is proposed to be the site within the visuomotor system where the nonlinear spatial-to-temporal transformation is carried out: the population encodes the intended saccade vector by its location in the motor map (spatial), and its trajectory and velocity by the distribution of firing rates (temporal). The neurons’ burst profiles vary systematically with their anatomical positions and intended saccade vectors, to account for the nonlinear main-sequence kinematics of saccades. Yet, the underlying collicular mechanisms that could result in these firing patterns are inaccessible to current neurobiological techniques. Here, we propose a simple spiking neural network model that reproduces the spike trains of saccade-related cells in the intermediate and deep SC layers during saccades. The model assumes that SC neurons have distinct biophysical properties for spike generation that depend on their anatomical position in combination with a center–surround lateral connectivity. Both factors are needed to account for the observed firing patterns. Our model offers a basis for neuronal algorithms for spatiotemporal transformations and bio-inspired optimal controllers.

## Introduction

Gathering high-definition visual information requires consecutive gaze shifts, as only the small foveal region in the central retina has a high visual resolution. The rapid step-like gaze shifts between points in the visual field are called *saccades*. Saccades are straight, extremely fast, goal-directed eye movements, which can reach peak velocities well over 1000 $^{\circ }$/s in monkey. They demonstrate remarkably stereotyped kinematic relationships, known as the “saccade main sequence” (Bahill et al. 1975): saccade duration increases approximately linearly with saccade amplitude, while peak eye velocity saturates for large saccade amplitudes. Further, the acceleration phase of saccades has a nearly fixed duration for all amplitudes leading to positively skewed velocity profiles (Van Opstal and Van Gisbergen 1987). These kinematic properties point at a nonlinearity in the system.

**Fig. 1:**
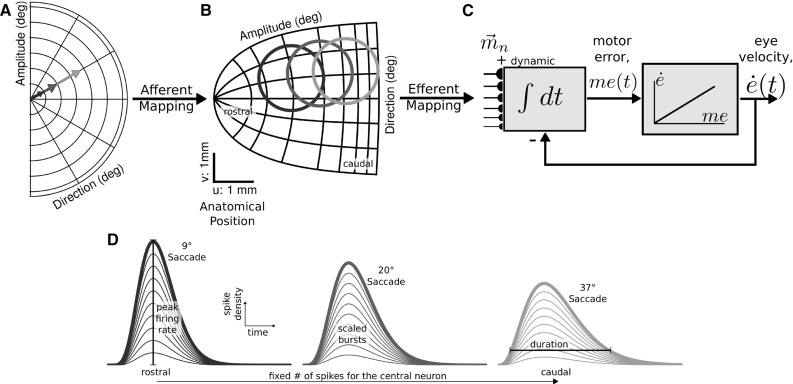
Afferent mapping of the right visual hemifield (**a**) results in the complex-logarithmic gaze motor map (**b**) that relates the anatomical position of active neural populations to saccade directions and amplitudes. Three saccade vectors in the visual field and anatomical positions of corresponding neural populations are highlighted. **c** Dynamic linear ensemble coding model can reproduce the saccade kinematics based on the SC spiking activity by the summation of a site-specific, fixed minivector ${\mathbf {m}}_n$ for each spike (Eqs. 1, 2). **d** Burst profiles and population activity characteristics within the SC for the three different saccade amplitudes shown in (**a**, **b**) (after Van Opstal and Goossens 2008)

These nonlinear kinematics could result from an optimal control mechanism, embedded in the neural pathways for saccade generation (Abrams et al. 1989; Harris and Wolpert 1998; Tanaka et al. 2006; Harris and Wolpert 2006; Van Beers 2008). The control overcomes the intrinsic signal-dependent noise within the visuomotor system to achieve an optimal speed–accuracy trade-off in line with Fitt’s Law (Fitts 1954; Van Opstal and Van Gisbergen 1989; Goossens and Van Opstal 2012). Consequently, the visuomotor system produces saccades with minimal end-point variability by moderating the speed of the movement as its amplitude increases.

The neural circuitry responsible for saccadic eye movements extends from the cerebral cortex to the pons in the brainstem. The midbrain superior colliculus (SC) is the final common terminal that specifies the vectorial eye displacement command for downstream oculomotor circuitry (Moschovakis et al. 1998) and could be in an excellent position to implement the optimal control principles, by mediating the sensorimotor transformations (Goossens and Van Opstal 2012). Indeed, recent evidence has also implicated a role for SC cells in specifying the nonlinear saccade kinematics (Goossens and Van Opstal 2006).

The SC contains an eye centered motor map that relates the anatomical location of the neural population to the intended movement vector (Ottes et al. 1986; Goossens and Van Opstal 2006). Each saccade command (Fig. 1a) is generated by an active Gaussian-shaped population (Fig. 1b), the location of which determines the saccade vector, whereas the temporal firing profiles of the neurons (Fig. 1c) have been shown to specify the saccade trajectory and kinematics. Small and large saccades are encoded by rostral and caudal populations, respectively. The SC output neurons exhibit bursting behavior in which the instantaneous firing rates reach up to 900 spikes/s, and saccade-related burst profiles have been characterized by positively skewed gamma functions (Van Opstal and Goossens 2008). The center of the population corresponds to the image point in the motor map of the saccade vector. Peak firing rate, burst duration and shape of the burst profile of the central neuron all depend systematically on the cell’s anatomical position in the map. The peak firing rates of neurons recruited for their optimal saccade decrease from rostral (small saccades) ($\sim$900 spikes/s) to caudal (large saccades) regions ($\sim$400 spikes/s), whereas burst durations increase accordingly (Fig. 1d).

We recently proposed that the neurons in the SC population encode an optimal, straight and fast trajectory of gaze shifts (Van Opstal and Goossens 2008) and revealed how each SC neuron is involved in different saccades (Goossens and Van Opstal 2012). In summary, SC neurons exhibit the following firing properties during saccades (schematized in Fig. 1d):

(i) each neuron in the motor map elicits a fixed number of spikes for its optimal (preferred) saccade;
(ii) a given neuron’s total spike count varies systematically with the saccade vector into its movement field;
(iii) all neurons in the population have similarly shaped (scaled and synchronized) temporal burst profiles during a saccade;
(iv) peak firing rate, burst duration and burst profile skewness of the central neuron in the population vary systematically across the motor map (Goossens and Van Opstal 2012).

According to the linear dynamic ensemble coding model (Fig. 1b, c), the saccade trajectory in two dimensions, ${\mathbf {S}}(t)$, can be decoded from the instantaneous spiking activity of the SC populations in the following way:

1 $$\begin{aligned} \mathbf {S}(t) = \sum _{n=1}^{N_{\mathrm{pop}}} \sum _{s=1}^{N_{\mathrm{spk}<\mathrm{t}}} \mathbf {m}_n \cdot \delta (t-\tau _{n,s}) \end{aligned}$$

with $\delta (t-\tau _{n,s})$, spike of neuron *n* at time $\tau _s$, weighted by a site-specific, fixed, minivector $\mathbf {m}_n$ (Fig. 1c). The latter is given by the efferent motor map (Ottes et al. 1986):

2 $$\begin{aligned} \mathbf {m}_n&= \kappa \Bigg [ A \Bigg (\exp \left( \frac{u_n}{B_u} \right) \cos \left( \frac{v_n}{B_v} \right) \nonumber \\&\quad \quad \qquad -1 \Bigg ), A \exp \left( \frac{u_n}{B_u} \right) \sin \left( \frac{v_n}{B_v} \right) \Bigg ] \end{aligned}$$

and thus fully determined by the location of a neuron in the motor map $[u_n, v_n]$. The SC map parameters $[B_u, B_v, A] = [1.4\,\hbox {mm},\,1.8\,\hbox {mm/rad}, 3{^\circ }]$; scaling factor $\kappa \lesssim 10^{-6}$ is specified by the assumed constant neural density in the motor map (Goossens and Van Opstal 2006; Van Opstal and Goossens 2008).

So far, most computational models of the SC have neglected the spike-level computations taking place in the motor map. One notable exception is the large-scale 7-layer spiking neural network scheme of Morén et al. (2013), which however, does not account for all the physiologically observed bursting properties of SC neurons. For instance, the synchronized firings of saccade-related neurons in the recruited population were neglected in that model [property (iii)].

In this study, we construct a biologically realistic, yet simple, spiking neural network model for ocular gaze shifts by the SC population to a single visual target. Our minimalistic model accounts for the experimentally observed dynamic transformations and the active representation of the saccade vector in the gaze motor map (Goossens and Van Opstal 2012). Spatiotemporal activity patterns of the SC motor map embody the nonlinear saccade kinematics, velocity profiles and eye displacement vector for optimal saccade trajectories (Van Gisbergen et al. 1985). Similarly, our SC model programs the saccadic motor commands by functionally acting as a nonlinear vectorial pulse generator. The resulting activity patterns of our model can be decoded according to the dynamic ensemble coding scheme of Eq. 1 by the downstream brainstem circuitry, which effectively acts as a linear local feedback loop (Fig. 1c). The construction of our model is constrained by the aforementioned firing properties of SC cells during saccades [listed above (i)–(iv)].

We hypothesize that these properties require:

- a location-dependent systematic tuning of the neuronal parameters that determine SC spike generation, and the profile of the intracollicular lateral connections, to account for properties (ii), (iii) and (iv);
- the input connections to the SC (from cortical sources) set the spike count properties across the population [properties (i) and (ii)].

Lateral interactions in the SC have been observed by anatomical (Behan and Kime 1996; Olivier et al. 1998) and electrophysiological (Munoz and Istvan 1998; Meredith and Ramoa 1998) studies, and they have been incorporated in several computational models of the SC motor map (Van Opstal and Van Gisbergen 1989; Trappenberg et al. 2001; Wang et al. 2012). Furthermore, we take the cortical input to the network to be translation invariant, encoding only the selected vector for a saccade target. A fixed input pattern is used to evoke network activity at varying locations in the SC map by topographic feedforward projections according to the afferent mapping. The network generates systematically varying responses at different locations. The temporal differences between burst responses encode the saccade kinematics.

Our model allows the investigation of SC activity as a sensorimotor interface performing spike-level computations that yield the dynamic saccade kinematics. Furthermore, since the model inherently adopts SC functionality, it offers a basis for neural algorithms for bio-inspired optimal control signal generators.

## Methods

### Network architecture

**Fig. 2:**
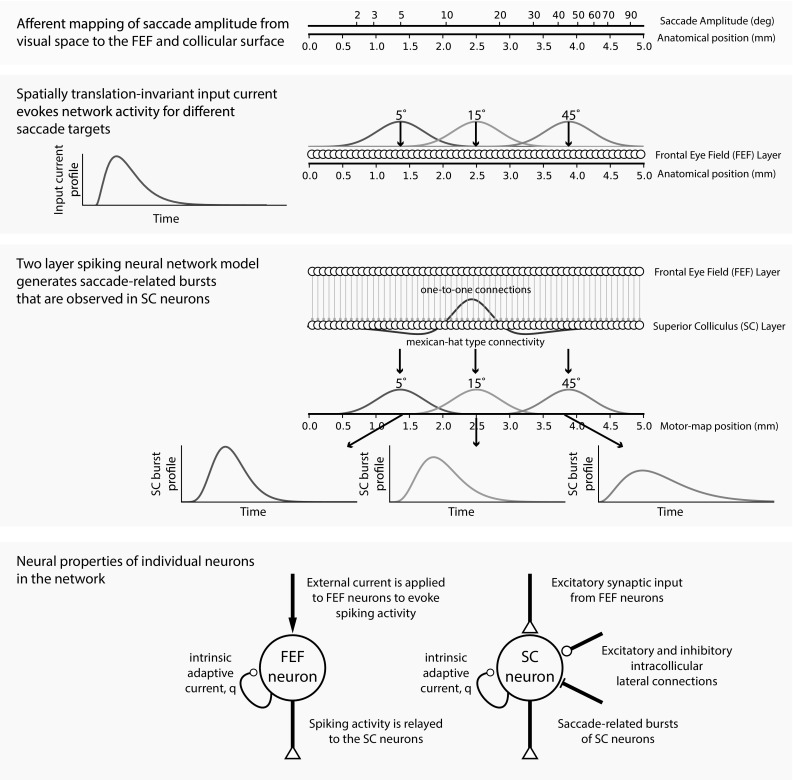
Schematic overview of the network scheme. Desired SC burst responses by central neurons in each population are generated after Van Opstal and Goossens (2008)

As a starting point, we constructed a one-dimensional spiking neural network with two layers (Fig. 2), representing frontal eye field (FEF) neurons (input layer) and gaze motor map neurons (SC layer), respectively. Each layer consists of 200 neurons uniformly distributed on a 5-mm straight line, which corresponds to the gaze motor map midline ($0^{\circ }$ direction). Thus, the network generates motor commands for horizontal saccades over a range of amplitudes from 0 to 104$^\circ$ (Eq. 3).

FEF neurons transform the external input current to spiking activity and relay their signals to the SC neurons through one-to-one, topography-preserving, connections. The SC neurons process the FEF spike trains with their topographically varying biophysical properties. Thus, the instantaneous responses of SC neurons to invariant FEF inputs become dissimilar at different locations within the gaze motor map, which encode saccade vectors of varying amplitudes.

### Log-polar mapping: visual space to neural coordinates

The afferent mapping translates a target point in visual space to the anatomical position of the center of the corresponding Gaussian-shaped population in both the FEF input layer and the SC motor map. It follows a log-polar projection of retinal coordinates onto Cartesian collicular coordinates (Ottes et al. 1986). In our one-dimensional network model, we only considered different saccade amplitudes in the same direction (amplitude *r*, and direction $\phi = 0^\circ$). The logarithmic mapping function determines the activation site of a saccade target, *T*, at eccentricity *r* on the 1D input layer $u_T$ by:

3 $$\begin{aligned} u_T = B_u \log {\left( \frac{r + A}{A} \right) } \end{aligned}$$

where $B_u = 1.4$ mm and $A = 3^\circ$ are the best-fit scaling factors for the monkey SC (Robinson 1972; Ottes et al. 1986) and determine size and shape of the gaze motor map.

### AdEx neuron model

We investigated the dynamics of the network model numerically in the Brian spiking neural network simulator (Goodman and Brette 2008). Simulations ran with 0.01 ms time steps. Brute-force search and genetic algorithms were used for parameter identification and network tuning since there exists no analytical solution for the system.

The neurons in the network are described by the adaptive exponential integrate-and-fire (AdEx) neuron model (Brette and Gerstner 2005) which accommodates bursting dynamics. The AdEx model is a conductance-based integrate-and-fire model with exponential membrane potential dependence. It reduces the Hodgkin–Huxley biophysical model to only two state variables: the membrane potential, *V*, and an adaptation current, *q*. The temporal dynamics of the system are given by the following differential equations for the the membrane potential and the adaptation current of neuron *n* respectively:

4 $$\begin{aligned} C \frac{\hbox {d}V_n}{\hbox {d}t}= & {} -g_{\mathrm{L}} (V_n-E_{\mathrm{L}}) + g_{\mathrm{L}} \eta \exp {\left( \frac{V_n-V_{T}}{\eta } \right) } \nonumber \\&- q_n+ I_{\mathrm{inp},n}(t),\end{aligned}$$

5 $$\begin{aligned} \tau _{q,n} \frac{dq_n}{\hbox {d}t}= & {} a(V_n-E_{\mathrm{L}}) - q_n , \end{aligned}$$

where *C* is the membrane capacitance, $g_{\mathrm{L}}$ is the leak conductance, $E_{\mathrm{L}}$ is the leak reversal potential, $\eta$ is a slope factor, $\tau _{q}$ is the adaptation time constant, *a* is the subthreshold adaptation constant and $I_{\mathrm{inp},n}$ is the total synaptic input current. All neural parameters are the same for input layer neurons. Thus, input-layer neurons have identical biophysical properties, and only receive an external input current $I_{\mathrm{inp},n} = I_{\mathrm{ext}}$ to evoke FEF activity. The two parameters that specify SC neurons: adaptation time constant, $\tau _{q,n}$ (location dependent), and synaptic input current, $I_{\mathrm{inp},n}= I_{\mathrm{syn},n}$ (location and activity dependent), however, vary systematically in the network. The remaining SC neural parameters; *C*, $g_{\mathrm{L}}$, $E_{\mathrm{L}}$, $\eta$, $V_T$ and *a* were tuned for neural bursting behavior (see Table 1 for the list and values of all parameters).

**Table 1:** Overview of all parameters used in the network simulations

*Input current*		
$\sigma _{\mathrm{pop}}$	0.5 mm	Recruited population size
$\beta$	0.03	Measure for burst duration
$\gamma$	1.8	Skewness and peak of the burst
$I_0$	3 pA	Scaling constant
*FEF neuron parameters*		
*C*	50 pF	Membrane capacitance
$g_{\mathrm{L}}$	2 nS	Leak conductance
$E_{\mathrm{L}}$	−70 mV	Leak reversal potential
$V_T$	−50 mV	Spike initiation threshold
$V_{\mathrm{peak}}$	−30 mV	Practical spiking threshold
$\eta$	2 mV	Spike slope factor
*a*	0 nS	Subthreshold adaptation
*b*	60 pA	Spike-triggered adaptation
$V_r$	−55 mV	Resting potential
$\tau _q$	30 ms	Adaptation time constant
*SC neuron parameters*		
*C*	280 pF	Membrane capacitance
$g_{\mathrm{L}}$	10 nS	Leak conductance
$E_{\mathrm{L}}$	−70 mV	Leak reversal potential
$V_T$	−50 mV	Spike initiation threshold
$V_{\mathrm{peak}}$	−30 mV	Practical spiking threshold
$\eta$	2 mV	Spike slope factor
*a*	4 nS	Subthreshold adaptation
*b*	80 pA	Spike-triggered adaptation
$V_r$	−45 mV	Resting potential
$\tau _q$	10–80 ms	Adaptation time constant (varies)
*SC synapse parameters*		
$E_e$	0 mV	Excitatory reversal potential
$E_i$	−80 mV	Inhibitory reversal potential
$\tau _e$	5 ms	Excitatory conductance decay
$\tau _i$	10 ms	Inhibitory conductance decay
$w^{\mathrm {F-S}}_{n}$	5–16 nS	Synaptic strengths (varies)
*Mexican hat parameters*		
$\bar{w}_{\mathrm{exc}}$	160 pS	Excitatory scaling factor
$\bar{w}_{\mathrm{inh}}$	50 pS	Inhibitory scaling factor
$\sigma _{\mathrm{exc}}$	0.4 mm	Range of excitatory synapses
$\sigma _{\mathrm{inh}}$	1.2 mm	Range of inhibitory synapses

Note that for $\tau _q$ and $w^{\mathrm {F-S}}_{n}$ the value ranges across the SC motor map coordinates are provided

Furthermore, the AdEx neuron model employs a smooth spike initiation zone instead of a strict spiking threshold. Once the membrane potential reaches the threshold value, $V_T$, the exponential term dominates and the membrane potential increases without bound. Even though a spike can theoretically occur when $V \rightarrow \infty$, we applied a practical spiking threshold $V_{\mathrm{peak}}$ for the time-driven simulations. For each spiking event at time, $\tau$, the membrane potential is reset to its resting potential, $V_{r}$, and the adaptation current, *q*, is increased by *b* to implement the spike-triggered adaptation:

6 $$\begin{aligned} V(\tau )\rightarrow & {} V_{r}\end{aligned}$$

7 $$\begin{aligned} q(\tau )\rightarrow & {} q(\tau ) + b . \end{aligned}$$

The neuron model has four free parameters (plus the input current) after rescaling the equations (Touboul and Brette 2008). Two of these parameters characterize the subthreshold dynamics: the ratio of time constants $\tau _q$/$\tau _m$ (with the membrane time constant $\tau _m=C/g_{\mathrm{L}}$) and the ratio of conductances *a*/$g_{\mathrm{L}}$. (*a* can be interpreted as the stationary adaptation conductance). Furthermore, the resting potential $V_r$ and the spike-triggered adaptation parameter *b* characterize the spiking patterns of the neuron (regular/irregular spiking, fast/slow spiking, tonic/phasic bursting, etc.).

### Saccade target representation: translation-invariant input current

We presented the desired saccade vector to the input layer by evoking a population activity centered around the site $u_T$, according to Eq. 3. Each neuron in the population received input current whereby the input current amplitudes depend on the distance of the neurons from the center at $u_T$. A spatial–temporal Gaussian-gamma function (Eq. 8) provides the input current to each neuron. Input-layer neurons transform the input current to spiking activity and relay to the SC neurons through topography-preserving one-to-one connections, which induces an SC population activity. We specified the translation-invariant input current profile to the FEF neurons as:

8 $$\begin{aligned} I_{\mathrm{ext}}(u_n, t) = I_{0} \exp \left( -\frac{ \Vert u_n-u_T \Vert ^2}{2\sigma _{\mathrm{pop}}^2}\right) t^{\gamma } \exp (-\beta t) \end{aligned}$$

where $u_n$ is the anatomical position of a neuron on the collicular map, $\sigma _{\mathrm{pop}}$ determines the size of the input population recruited for a saccade, *t* is time, $I_0$ is a constant scaling factor. Time-dependent terms characterize the temporal activity profile by $\gamma$ and $\beta$. The spatial Gaussian function (position, $u_n$) scales the temporal current profile by the distance from the FEF population center.

### The SC synapse model

The total synaptic input current for an SC neuron is governed by the spiking activity of the input-layer neurons and conductance-based synapses:

9 $$\begin{aligned} I_{\mathrm{syn},n}(t) = g^{\mathrm{exc}}_n(t)(E_e-V_n(t)) + g^{\mathrm{inh}}_n(t)(E_i-V_n(t)) \end{aligned}$$

where $g^{\mathrm{exc}}$ and $g^{\mathrm{inh}}$ are excitatory and inhibitory conductances, $E_\mathrm{e}$ and $E_\mathrm{i}$ are excitatory and inhibitory reversal potentials, respectively. These conductances increase instantly for a presynaptic spike by a factor of synaptic strength between neurons and decay exponentially otherwise, following:

10 $$\begin{aligned} \begin{aligned} \tau _{\mathrm{exc}} \frac{\hbox {d} g_n^{\mathrm{exc}}}{\hbox {d}t} =&-g_n^{\mathrm{exc}} + \tau _{\mathrm{exc}} w^{\mathrm {F-S}}_n \sum _s^{N^{\mathrm{FEF}}_{\mathrm{spk}}} \delta (t-\tau _{n,s}) \\&+ \tau _{\mathrm{exc}} \sum _i^{N^{\mathrm{SC}}_{\mathrm{pop}}} w^{\mathrm{exc}}_{i,n} \sum _s^{N^{\mathrm{SC}_i}_{\mathrm{spk}}} \delta (t-\tau _{i,s}) \\ \tau _{\mathrm{inh}} \frac{d g_n^{\mathrm{inh}}}{\hbox {d}t} =&-g_n^{\mathrm{inh}} + \tau _{\mathrm{inh}} \sum _i^{N^{\mathrm{SC}}_{\mathrm{pop}}} w^{\mathrm{inh}}_{i,n} \sum _s^{N^{\mathrm{SC}_i}_{\mathrm{spk}}} \delta (t-\tau _{i,s}) \end{aligned} \end{aligned}$$

with $\tau _{\mathrm{exc}}$ and $\tau _{\mathrm{inh}}$, the excitatory and inhibitory time constants; $w^{\mathrm {F-S}}_n$, the synaptic strengths between two layers; $w^{\mathrm{exc}}_{i,n}$ and $w^{\mathrm{inh}}_{i,n}$ intracollicular excitatory and inhibitory lateral connection strengths, from neuron *i* to *n*, respectively, and $\tau$, the spike timing of presynaptic FEF ($\tau _{n,s}$) and SC ($\tau _{i,s}$) neurons.

With conductance-based synaptic connections, spike propagation occurs in a biologically realistic way since the postsynaptic projection of a presynaptic spike is dependent on the membrane voltage of the postsynaptic neuron. In this way, the state of a neuron determines its susceptibility to presynaptic spikes.

### Lateral connections

We hypothesize that the observed synchronization of bursts of saccade-related neurons in the population arises from lateral interactions between SC neurons. We incorporated a “Mexican Hat”-type lateral connection scheme in the model, where the net synaptic effect is given by the difference between two Gaussians (e.g., (Trappenberg et al. 2001; Eqs. 11, 12). Accordingly, neurons are connected with strong short-range excitatory and weak long-range inhibitory synapses, which implements a dynamic soft winner-take-all (WTA) mechanism: not only one neuron remains active, but the “winner” affects the activity of the other active neurons. The central neuron governs the population activity, since it is the most active one in the recruited population. As a result, all recruited neurons exhibit similarly shaped bursting profiles as the central neuron.

Two Gaussians describe the excitatory $w^{\mathrm{exc}}_{i,n}$ and inhibitory $w^{\mathrm{inh}}_{i,n}$ connection strengths between collicular neurons based on their spatial separation:

11 $$\begin{aligned} w^{\mathrm{exc}}_{i,n}= & {} \bar{w}_{\mathrm{exc}} \exp {\left( -\frac{\Vert u_i - u_n \Vert ^2}{2 \sigma _{\mathrm{exc}}^2}\right) } \quad \text {for } n \ne i \end{aligned}$$

12 $$\begin{aligned} w^{\mathrm{inh}}_{i,n}= & {} \bar{w}_{\mathrm{inh}} \exp {\left( -\frac{\Vert u_i - u_n \Vert ^2}{2 \sigma _{\mathrm{inh}}^2}\right) } \quad \text {for } n \ne i \end{aligned}$$

with $\bar{w}_{\mathrm{exc}} > \bar{w}_{\mathrm{inh}}$ and $\sigma _{\mathrm{inh}} > \sigma _{\mathrm{exc}}$. Self-projections are omitted to prevent neural activity from blowing up:

13 $$\begin{aligned} w^{\mathrm{exc}}_{i,i} = w^{\mathrm{inh}}_{i,i} = 0. \end{aligned}$$

### Cross-correlation analysis

To quantify similarity between burst profiles of neurons at different locations within the population, we computed the cross-correlation between the burst profiles of the central neuron, $P_{\mathrm{cntr}}(t)$, with other neurons along the rostral-to-caudal direction from the center, $P_n(t)$. In this analysis, we considered a time window from 10 ms before to 40 ms after the saccade onset ($t=0$) for each cell. The cross-correlation was calculated after all burst profiles were first normalized with respect to their own peak firing rate:

14 $$\begin{aligned} r_n = \frac{\sum _t \widehat{P}_{\mathrm{cntr}}(t) \cdot \widehat{P}_n(t)}{\sqrt{\sum _t \widehat{P}^2_{\mathrm{cntr}}(t)} \cdot \sqrt{\sum _t \widehat{P}^2_n(t)}} \quad \mathrm {with} \quad \widehat{P}=\frac{P}{\max {(P)}}.\nonumber \\ \end{aligned}$$

We restricted our cross-correlation analysis to the population activity within 0.65 mm from the center since the firing rates for cells at larger distances rapidly dropped to zero. Spike density is computed by convolution of a spike train with a Gaussian kernel (width 5 ms).

### Identification of lateral connectivity parameters

For each saccade amplitude, the recruited population size is the same. The widths of the Mexican hat connectivity ($\sigma _{\mathrm{inh}}$ and $\sigma _{\mathrm{exc}}$) are determined based on the size of this active population, because these parameters govern the spatial range of a neuron’s spike influence in the network. The widths are fixed and large enough to yield local excitation and global inhibition. Connection strengths ($\bar{w}_{\mathrm{exc}}$ and $\bar{w}_{\mathrm{inh}}$), on the other hand, affect spiking behavior and local network dynamics. These values affect how much excitation and inhibition each single neuron will receive from and project to others based on the ongoing activity. Thus, the numerical values of these parameters depend on the parameters of single neurons. Strong excitation would result in a spread of population activity, whereas a strong inhibition would fade out neural activity altogether. Thus, balanced excitation and inhibition is required to establish an active Gaussian population.

To find suitable parameters for the lateral connection strengths, we used a genetic algorithm. In this algorithm, an initial set of 10 $\bar{w}_{\mathrm{exc}}$ and $\bar{w}_{\mathrm{inh}}$ pairs are generated randomly as candidate solutions. This set is considered as the first generation in the genetic algorithm. The network simulations with each pair generated population activity patterns for seven different saccade amplitudes (selected as $r =$ [2, 5, 9, 14, 20, 27, 35]$^\circ$). Candidate solutions are subsequently ranked with the fitness function (Eq. 15). Based on their ranks, the two best-fit candidates are chosen as *elites* and transferred directly to the next generation with 8 new solution candidates, *children*. Each of these children is generated from a randomly picked pair of *parents* from the pool of 6 best-fit candidates in the previous generation. The same parent pair is not used to produce more than one child. A child is produced by a random crossover point over a modular representation of parent pair and 5% mutation probability. This procedure is repeated until 2 best-fit individuals ranked the same in successive generations.

The genetic algorithm minimized the root mean squared errors (RMSE) between the spiking network responses and the rate-based model of Van Opstal and Goossens (2008): from the fitness evaluation for each generation, we calculated RMSE between the peak firing rates and the number of elicited spikes from the central cells. Furthermore, the cross-correlations between all active neurons and the central cell are taken into account. This assured that the gaze motor map characteristics are taken into account for the parameter identification. The fitness function is defined with a weighted RMSE summation;

15 $$\begin{aligned} \hbox {Fitness}= & {} 10^{-1} \times \hbox {RMSE}(F_{\mathrm{peaks}}) \nonumber \\&+\,10^1 \times \hbox {RMSE}(\# \, \hbox {of} \, \hbox {spikes}) \nonumber \\&+\,10^3 \times \hbox {RMSE} (\hbox {cross} \, \hbox {correlation}) \end{aligned}$$

where the weights are empirically chosen to similar ranges since the $F_{\mathrm{peaks}}$ vary from roughly 750 spikes/s to 430 spikes/s, the number of spikes varies between 18 and 22, and the cross-correlation values are below 1.

**Fig. 3:**
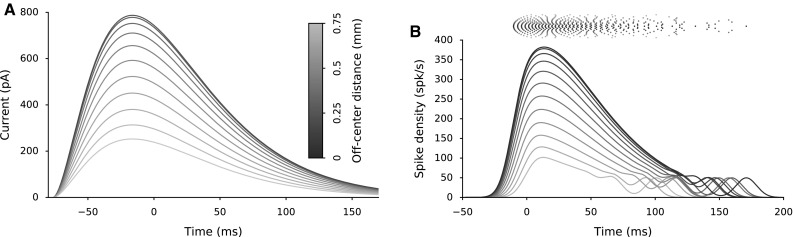
**a** Input current, $I_{\mathrm{ext}}$, to FEF layer neurons. **b** Spike trains and spike densities of FEF layer neurons in response to $I_{\mathrm{ext}}$. Spike densities are calculated with a 8 ms gaussian kernel

Peak firing rates of the central neurons from each populations are calculated by convolving the spike trains with a gaussian kernel (with 8 ms kernel width). RMSE values for $F_{\mathrm{peaks}}$ were calculated by applying the firing rate model values;

16 $$\begin{aligned} F_{\mathrm{peak}}(r) = \frac{F_0}{\sqrt{1+\beta r}} \end{aligned}$$

where $F_0 =$ 800 spikes/s and $\beta =$ 0.07 ms/$^\circ$ (Van Opstal and Goossens 2008). RMSE of total spike counts from central cells were calculated with respect to $N = 20$ spikes, independent of the saccade vector or neuron position. Synchrony of neural activity was calculated as the RMSE of deviations from 1 for the cross-correlations between the central cell and all other active cells in the population Eq. 14.

### Generation of eye movements

Eye movements are generated by the population activity following the linear ensemble coding model (Eq. 1). The one-dimensional efferent motor map was calculated by Eq. 2 for $v_n = 0$. For any network configuration throughout this paper, scaling factor of the efferent motor map ($\kappa$ in Eq. 2) is calibrated for 21$^\circ$ saccade. Resulting eye displacement, $\mathbf {S}(t)$ is then interpolated with first order spline for equidistance time points. Finally, the interpolation is smoothed with a Savitzky–Golay filter to compute the derivative, the eye velocity.

## Results

### Input current evokes spiking activity of FEF layer neurons

Table 1 summarizes the list of parameters of the neurons in the two-layer network. Figure 3a illustrates the input current, $I_{\mathrm{ext}}$ (Methods 2.4, Eq. 8), acting on FEF layer neurons and the resulting spiking response of FEF neurons (Fig. 3b) for any saccade target for the chosen parameter values in Table 1. For illustration purposes, only a set of uniformly distributed FEF neurons (including the central neuron) is shown. Spike density functions of FEF layer neurons reflect the input current properties; all neurons have scaled spike densities, which decrease as the distance from the central neuron increases. These spike trains impinge onto SC neurons with one-to-one connections (Fig. 2).

### Bursting mechanism of AdEx neuron model

**Fig. 4:**
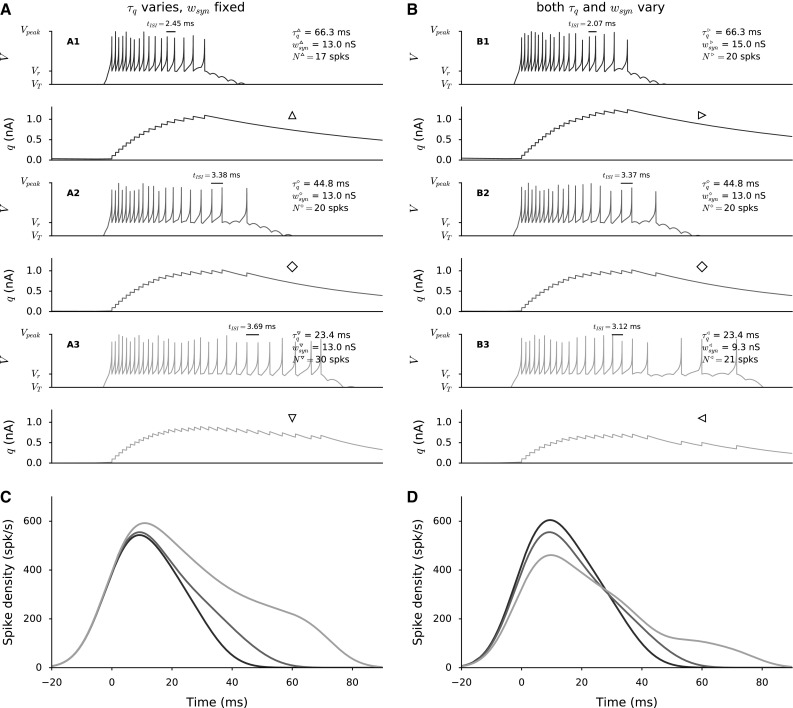
Effect of adaptive characteristics of an AdEx neuron on the evoked neural activity by the input pattern of Fig. 3b. Temporal evolutions of the state variables: membrane potential, *V*, and adaptive current, *q*, for varying adaptation time constants, $\tau _q$ for fixed synaptic input strengths (**a**) and for varying synaptic input strengths (**b**) Spike density functions of the burst profiles for fixed synaptic input strengths (**c**) and for varying synaptic input strengths (**d**). Spike densities are calculated with a 8 ms gaussian kernel

**Fig. 5:**
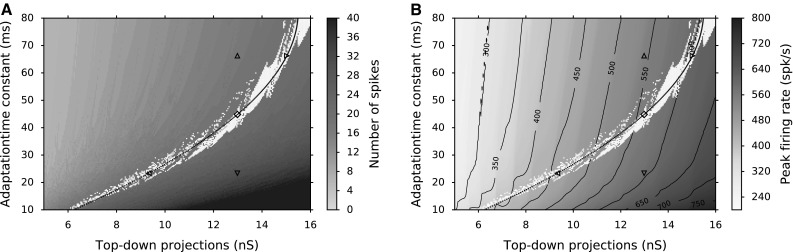
Brute-force parameter search for the adaptation time constant $\tau _q$ and top-down synaptic projections from FEF to SC layers $w_{n}^\mathrm {F-S}$. Single AdEx neurons configured with SC parameters are driven by the most active neuron in the FEF population (Eq. 8). **a** Total number of spikes in the burst. **b** Peak firing rate of the burst profile. White points: the neurons emit 20 spikes in their burst, and the contours show the peak firing rates. *Black lines* depict the parameter values used for SC neurons in the network simulations. They are calculated by a second-order polynomial regression of $\tau _q$ and $w_{n}^\mathrm {F-S}$ for 20 spikes in burst. Behavior of AdEx neuron at points $^{\vartriangle ,\triangledown ,\triangleleft ,\triangleright }$ and $^\diamond$ are illustrated in Fig. 4a, b

To illustrate the effect of the relevant neuronal parameters on the response behavior of the AdEx neuron model, Fig. 4a, b shows the temporal evolution of the two state variables, membrane potential, *V*(*t*), and adaptive current, *q*(*t*), for different sets of parameter values. Figure 4a displays the neural responses for three adaptation time constants and a fixed synaptic input strength (identified by symbols $\vartriangle$, $\diamond$ and $\triangledown$ in Fig. 5), whereas in Fig. 4b the synaptic strengths vary too (indicated by $\triangleleft$, $\diamond$ and $\triangleright$ in Fig. 5). The same presynaptic spike train (the peak trace shown in Fig. 3b) impinges on all six illustrated neurons. Thus, the conductance is the same for the three cases in Fig. 4a since the synaptic strengths are fixed (see Methods 2.5). However, the total number of spikes and burst profiles vary in these three cases since the adaptation current affects the susceptibility of a neuron to incoming synaptic conductance. The differences between responses result from varying the adaptation time constant, $\tau _q$. For fixed synaptic connection values (Fig. 4a), higher adaptation time constant results in fewer spikes, $N_{\mathrm{spk}}$, and a lower peak firing rate (dark blue in Fig. 4c) because *q* reaches high values faster (*q* reaches 1 nA in Fig. 4A1 earlier than A2 and A3). This effect results from a fast adaptive current buildup by each consecutive spike in a burst. For lower $\tau _q$ values (Fig. 4A3), the adaptation decay is faster; *q* decays fast enough to let the next spike occur earlier in the burst. Spike-triggered adaptation in the model is implemented by an instant increase of the intrinsic adaptation current variable, *q*, which is increased by *b* with each spike (Eq. 6). More importantly, $\tau _q$ affects the inter-spike intervals (ISIs) in these bursts, especially after the peak firing of the bursts; ISIs between consecutive spikes in the burst increase systematically as $\tau _q$ decrease (Fig. 4A3), resulting in the longer tails of the burst profiles (Fig. 4c).

In Fig. 4b, synaptic connection strengths, $w_n^{\mathrm{F-S}}$, vary as well. Thus, the total excitatory input current acting on these neurons varies for the identical presynaptic spike trains (Fig. 4B1, B2 and B3). For suitable parameter settings, the number of spikes in the bursts is fixed. A strong adaptation current acting on a neuron with high $\tau _q$ is compensated by an increased conductance through higher synaptic connection strength (B1). On the other hand, a decreased total input current for the fast decaying adaptive current (B3) results in fewer spikes. Varying ISIs results in dissimilar burst profiles (shown in Fig. 4d), both in their peaks and burst durations. Lower peak firing rates are accompanied with longer burst tails, since the number of spikes in the bursts is approximately fixed.

### Parameter tuning for spatial variation of SC burst profiles

To find suitable parameters for the biophysical properties of SC neurons, we performed a brute-force search procedure. The SC neurons had fixed parameters, except for their adaptation time constants, $\tau _q$, and top-down projections from FEF to SC layer neurons, $w_{n}^\mathrm {F-S}$. The fixed parameters for two types of neurons that generate spiking activity of FEF layer neurons and SC bursting behavior are given in Table 1. By varying the adaptation time constant, $\tau _q$, the decay speed of the adaptation current, *q*, could be varied, which accounts for the systematic changes in behavior of SC cells as function of their location in the map. Systematic changes in top-down projections, $w_{n}^\mathrm {F-S}$, can compensate for the varying input sensitivity of the neurons resulting from varying adaptive properties and hence keep the number of emitted spikes constant (as in Fig. 4b). To illustrate the burst properties of the AdEx model neurons, Fig. 5 shows the total number of emitted spikes (A) and the peak firing rate (B) of the burst for different $\tau _q$ and $w_i^\mathrm {F-S}$ values, when driven by the same input spike train. It is seen that higher $w_i^\mathrm {F-S}$ and lower $\tau _q$ values result in more spikes and higher peak firing rates, whereas lower $w_i^\mathrm {F-S}$ and higher $\tau _q$ values result in fewer spikes and lower peak firing rates. The parameter pairs resulting in 20 spikes in the burst are highlighted (white color in Fig. 5).

Figure 5b shows how the peak firing rates (contours) change for the parameter pairs while the total number of spikes in the burst stays fixed (white dots represent 20 spikes in a burst). These analyses lead to a selection of $\tau _q$ and $w_{n}^\mathrm {F-S}$ pairs that correspond to observed burst properties in the gaze motor map. The total number of spikes in the burst remains constant as the peak firing rate drops from the rostral to caudal zone. Thus, we fitted the parameter pairs that yielded 20 spikes in the burst with a second-order polynomial (black curve in Fig. 5). The fitted values were used in the network simulations to set up the gaze motor map characteristics of our model.

**Fig. 6:**
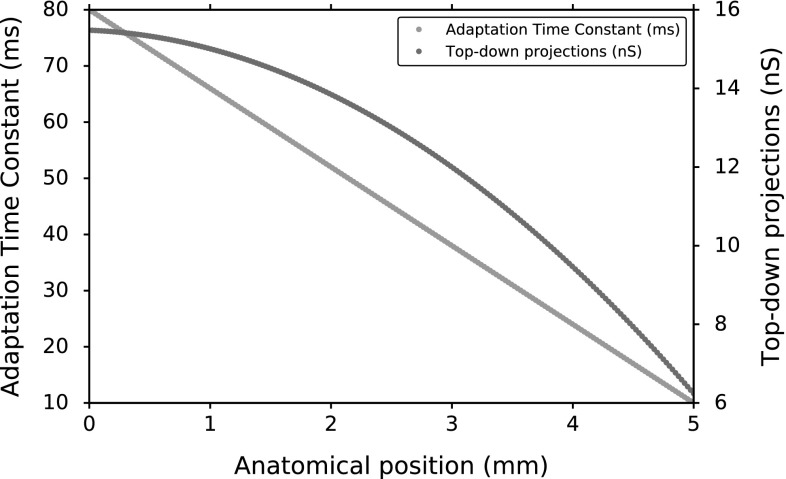
Position-dependent values of $\tau _q$ and $w_{n}^\mathrm {F-S}$ as used in the network simulations to set up spatial variation in the neural activity patterns

Figure 6 shows the position-dependent values of $\tau _q$ and $w_{n}^\mathrm {F-S}$ used in the network simulations as a function of the anatomical position. The adaptation time constants were chosen to decrease linearly along the SC map from rostral to caudal locations (green line). The corresponding values for the synaptic strengths were then calculated with the second-order polynomial fit of Fig. 5. In that way, each SC neuron had distinct biophysical properties and their burst profiles varied systematically along the gaze motor map midline.

**Fig. 7:**
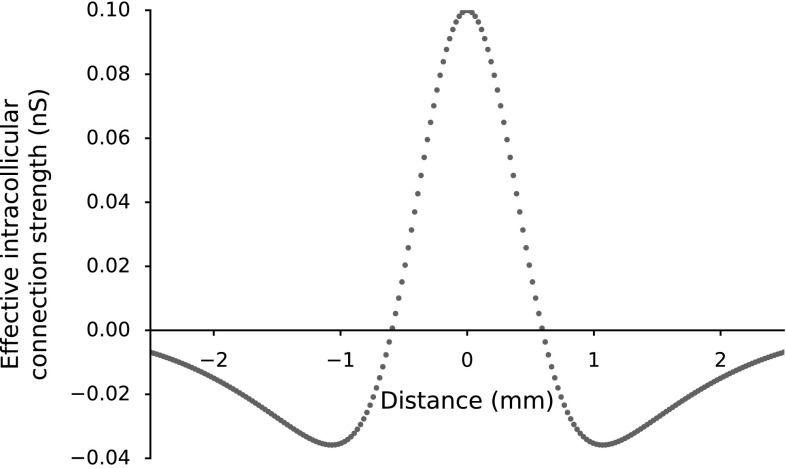
Difference between excitatory and inhibitory intracollicular synaptic projections constructs a Mexican hat-type center–surround interaction within the SC. Wider inhibitory connections width ($\sigma _{\mathrm{inh}}= 1.2$ mm > $\sigma _{\mathrm{exc}}= 0.4$ mm) with larger excitatory connection weight ($\bar{w}_{\mathrm{exc}}= 160$ pS $> \bar{w}_{\mathrm{inh}} = 50$ pS results in local excitation and global inhibition. $\bar{w}_{\mathrm{exc}}$ and $\bar{w}_{\mathrm{inh}}$ values are optimized by a genetic algorithm to minimize burst profile dissimilarities (Eqs. 12, 11). It thus accounts for the synchronization of burst profiles within the population

Figure 7 depicts the net intracollicular lateral connection strengths from each neuron as obtained from the genetic algorithm. Lateral connections yield short-range excitatory and long-range inhibitory effects of each neuron. Effectively, SC neurons have both excitatory and inhibitory projections among them with different time constants and reversal potentials (summarized in Table 1). However, the differences in the synaptic strengths display a center–surround antagonism yielding a Mexican hat type of lateral connections.

**Fig. 8:**
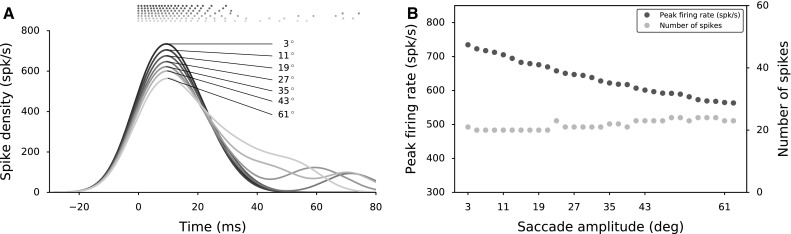
**a** Spike trains and burst profiles for central cells in populations for different saccade amplitudes show increasing burst durations. Burst profiles are aligned to $t=0$ ms at the first elicited spike, and thus, the eye movement onset. Spike densities are calculated with a 8 ms gaussian kernel. **b** Number of spikes emitted by the central cell is roughly constant between 20 and 23 spikes. The peak firing rate of the central cell decreases markedly from approximately 750 spikes/s to 550 spikes/s as the saccade amplitude increases from 3$^{\circ }$ to 63$^{\circ }$

### Central neuron and optimal saccade vector

A proper selection of $\tau _{q,n} - w_{n}^\mathrm {F-S}$ parameter pairs along the rostral-to-caudal axis ensures burst profiles that reflect experimentally observed spatial variations in the SC motor map. When these neurons are recruited for their optimal saccades, rostral neurons exhibit higher peak firing rates in their bursts and shorter durations [property (iv)] when compared to caudal neurons. Figure 8a shows the simulated spike trains and burst profiles for several SC cells along the motor map when they are recruited for their optimal saccade. The temporal profiles of the bursts display a systematic variation of burst duration, skewness and peak firing rate. The peak firing rates decrease from 750 to 550 spikes/s as the saccade amplitude increases from 3 to 63$^\circ$ [property (iv), Fig. 8b] and the spike counts of the cells stay roughly constant, varying nonsystematically between 20 and 23 spikes [property (i)]. Note that although these network simulations incorporate lateral interactions, the characteristics of central cell bursts are mostly due to the position-dependent distinct properties of SC cells.

### Synchronized population activity of recruited neurons

The burst profiles of distinct motor map neurons do not solely depend on their anatomical positions but also on the saccade vector for which they are recruited. If the SC were to act as an optimal controller for saccades, the neurons should synchronize their burst profiles so that the net command signal could dynamically reflect a straight trajectory with scaled and optimal vertical and horizontal velocity components. Accordingly, all neurons within the recruited population should exhibit burst profiles that are scaled versions of one another [property (iii)].

**Fig. 9:**
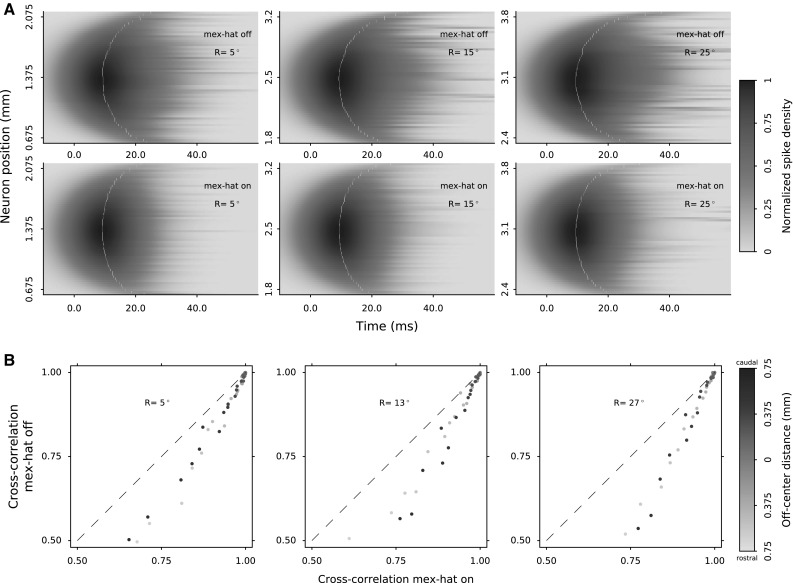
**a** Lateral connections synchronize the burst profiles of the neurons in a recruited population. Simulation results without lateral connections (*top row* in **a**) display poorer network performance compared to the synchronized activity via lateral connections (*bottom row* in **a**). Population activities are normalized by the peak firing rate of the central cell in each population. The peak firing moments are marked to highlight improved temporal aligning via lateral interactions, especially in the population centers. **b** Cross-correlation of the burst profiles of the central neuron with the other recruited neurons. Each data point depicts cross-correlations between the neuron pair with and without lateral connections. Neuron’s distance to the population center is *color-coded*. *Dashed lines* depict the diagonal unity line. The points below the dashed lines are in favor of lateral connections. Note that this comparison is possible when the lateral connections do not affect the size and total spike counts of the active populations (shown in Fig. 10)

Lateral connections with a Mexican hat shape accounts for this observation (Fig. 7). Figure 9a displays bursting profiles of three neural populations in the motor map that encode saccades of amplitudes $5^\circ$, $15^\circ$ and $25^\circ$, respectively. The upper panels depict the simulated population activity of a one-to-one network, without lateral connections. The lower panels display the effect of active lateral connections on the bursting profiles. Note that the lateral connections set up a soft winner-take-all mechanism, in which the central neurons dictate their bursting profiles to the other neurons in the population.

**Fig. 10:**
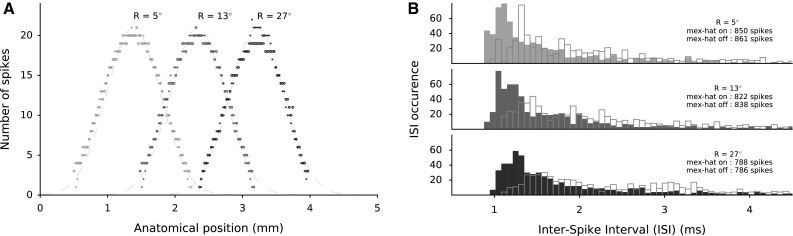
**a** Spike counts of each recruited neuron for three different saccade vectors with and without lateral connections are depicted by solid dots and open circles, respectively. Gaussian curves are plotted in dashed lines only to illustrate similarities between active populations. They are centered around the central cell of each population with a fixed width of $\sigma =0.4$ mm and a scaling factor of 21 spikes. **b** ISI distributions of the spike trains from all neurons are shown in three active populations with (*filled bars*) and without (*hollow bars*) lateral interactions. The total number of spikes in each population is comparable, whether lateral connections are included or not. The shift to longer ISI’s for caudal populations results in longer burst durations and lower firing rates when larger eye movements are encoded

**Fig. 11:**
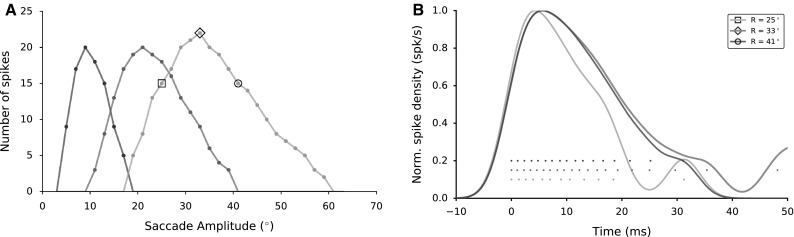
**a** Spike counts of the SC neurons in response to different saccade amplitudes determine their movement fields. Preferred saccade amplitudes: $9^{\circ }$, $21^{\circ }$ and $33^{\circ }$, respectively. Spike counts decrease as the contributed saccade diverges from the preferred saccade of the neuron. Note that caudal neurons have broader tuning compared to rostral neurons. That property is a result of the exponential nature of the efferent mapping function. **b** Burst profiles of one neuron, in response to three different saccade amplitudes: $25^{\circ }$, $33^{\circ }$ (its preferred saccade), and $41^{\circ }$. To emphasize burst profile differences, spike trains are convolved with a Gaussian kernel of 3 ms width, normalized by their peaks and aligned to the first spikes for each at $t=0$ ms

Lateral connections correct for the dissimilarities in cell-burst properties arising from the distinct biophysical properties and synaptic strengths. Note that the latencies of peak firing, as well as the variability in burst skewness within the population, decrease substantially for the simulations with lateral connections. The net effect of the lateral connections is local excitation and surrounding inhibition from each neuron to the neurons in its periphery. Thus, the closer neurons, by exciting each other, are synchronizing their burst profiles. Note that the overall burst durations decrease when the lateral connections are included. This results in an increase of the peak firing rates within the population. The effects of lateral interactions are also apparent in the ISI distributions of the activated neurons in the population (Fig. 10b). Furthermore, the accumulated inhibition in the network kicks in and affects the burst skewness’ of the neurons after peak firing. This results in more similar burst profiles within the population.

As a quantitative measure of similarity, we computed the cross-correlation of all burst profiles with the central neuron in each population. Figure 9b displays how lateral connections affect the cross-correlations between the burst profiles of the central neuron and other active neurons in each population. The cross-correlations are naturally high since all firing rates resemble gamma-bursts. However, lateral connections increase the similarity between the burst profiles, and thus, all data points lie further from the diagonal.

### Spatiotemporal burst dynamics of recruited neurons

Each saccadic motor command is generated by an active Gaussian population. The most active neuron in a recruited population is the central neuron. It elicits the largest number of spikes in the population and exhibits the highest peak firing rate. The number of spikes elicited by the other neurons decrease with distance from the central cell, both in caudal and rostral directions. Figure 10a displays the spike counts of each neuron in the gaze motor map for three different saccade commands with and without lateral connections. Figure 10a captures some important properties that are related to the gaze motor map: First, a neuron contributes to many different saccade vectors with a different number of spikes described by its movement field (Fig. 11). Second, the total number of neurons contributing to different saccade vectors is roughly fixed. Since the neurons are uniformly distributed, the widths of the Gaussian populations are the same. Third, the total number of spikes emitted by each population is constant. As such, the number of spikes elicited by the neurons reflects the spatially translation-invariant afferent target encoding scheme as suggested by Ottes et al. (1986). Furthermore, the size of the active population and total spike counts remain unaffected when the lateral interactions are included.

However, the temporal characteristics of the bursts do vary with the cell’s locations in the motor map and with lateral interactions. Figure 10b shows the ISI histograms for all recruited neurons in the three populations (of panel A). For larger saccade amplitudes, the ISI distribution shifts toward longer intervals. This property reflects the lower firing rates in the spike trains of caudal cells and results from the increased durations of the bursts for the same total number of spikes. Figure 10 summarizes how lateral interactions affect the temporal dynamics of neural firings, rather than the spatial characteristics of the recruited populations.

### Saccade-dependent burst profiles of SC neurons

The spike count for a given neuron varies systematically with the saccade vector into its movement field [property (ii), Fig. 10]. Figure 11a exhibits the spike counts for three neurons in response to varying saccade vectors. The optimal saccade vectors for these three neurons are obtained for the highest number of spikes. Thus, the preferred saccade amplitudes are $\sim 9^{\circ }$, $21^{\circ }$ and $33^{\circ }$, respectively. Spike counts decrease systematically as the saccade amplitude differs from the preferred saccade amplitude of the neuron. Further, in the spike counts of the three neurons for various saccade vectors, the log-polar characteristics of the gaze motor map are also apparent. Caudal neurons have a much wider movement field than rostral cells.

**Fig. 12:**
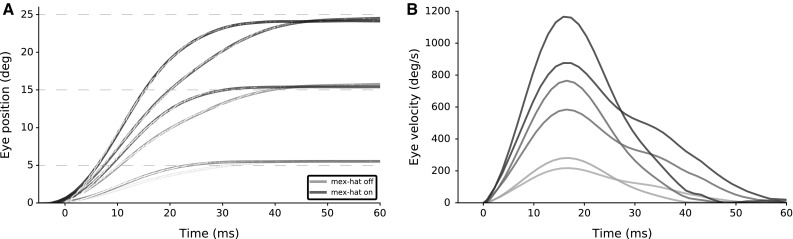
**a** Three eye movements (to saccade targets: 5, 15, 25 degrees) are shown for two cases: with (*blue*) and without (*green*) lateral interactions among SC neurons (the associated population activities shown in Fig. 9). Eye traces were calculated as a weighted dynamic sum of the elicited population spikes, which are visible as *white dots* in the eye position traces. Interpolation and smoothing of these data points yield the emerging eye position traces that allow computation of the associated velocity profiles (see Methods 2.9). **b** Eye velocity profiles show the strong effect of the lateral connections on saccade performance. Note also that the peak eye velocities increase with saccade amplitude for each population

A neuron’s burst profile, when recruited for different saccade vectors, will also be dissimilar. Figure 11b depicts the normalized firing rates of the neuron with the preferred saccade amplitude $33^{\circ }$ when it is recruited for three different saccade amplitudes (highlighted in Fig. 11a by the three symbols): its preferred saccade amplitude ($33^{\circ }$, diamond), a smaller ($25^{\circ }$, square) and a larger ($47^{\circ }$, circle) saccade for which the neuron contributed the same number of spikes. The neuron’s burst profiles are quite different for saccades into its movement field, even when it emits the same number of spikes. The neuron’s spike density decreases faster when it is recruited for the smaller saccade vector, than for a larger one. A direct comparison between these responses and the response profile to the optimal saccade vector is not possible, since it emits more spikes for its optimal saccade vector. Even so, the three saccade burst profiles for the three saccades have different shapes. Hence, the burst shape is not dictated by the location of the cell within the motor map, but by the saccade for which it is recruited. This property results from the lateral interactions among SC cells.

### Eye movements generated by the spiking population

Eye movements are constructed by the linear ensemble coding model with spiking neurons, as a dynamic weighted sum of the SC population spikes (Eqs. 1, 2). The trajectories and velocity profiles of three saccades are depicted in Figure 12. These are the resulting eye movements of three population activities shown in Figure 9. Eye positions show that the population activity results in on target saccades (Fig. 12a). Eye kinematics, on the other hand, differs and synchronized bursts result smoother and more realistic eye movements. Computed eye velocities (Fig. 12b) display that the lateral interactions result in higher peak eye velocities (since the synchronized bursts are integrated dynamically) and that the eye decelerates steadily until the target is reached. Note that the inclusion of lateral interactions results in increased firing rates, synchronized bursts and much faster saccades.

### Characterization of lateral interactions

The network is tuned to generate activity patterns that are observed in measured saccade-related SC cells. The topographic map and location-dependent firing properties are imposed by site-specific biophysical neural parameters ($\tau _q$ and $w_{n}^\mathrm {F-S}$). Topographical activity properties such as population spike count, number of recruited neurons, spike count of the central neuron and peak firing gradient along the rostral–caudal axis are determined by these biophysical parameters. On the other hand, synchronized population activity is regulated by lateral interactions among neurons, leading to optimized saccade performance. Clearly, also the lateral interaction profiles need to be precisely tuned in order to achieve optimal motor control. These are essentially two free parameters to uniquely define the Mexican hat profiles, which we here take as the width and depth of the inhibitory connections. Varying the spatial extent and strength of excitatory and inhibitory connections results in different population activity profiles and eye movement trajectories.

**Fig. 13:**
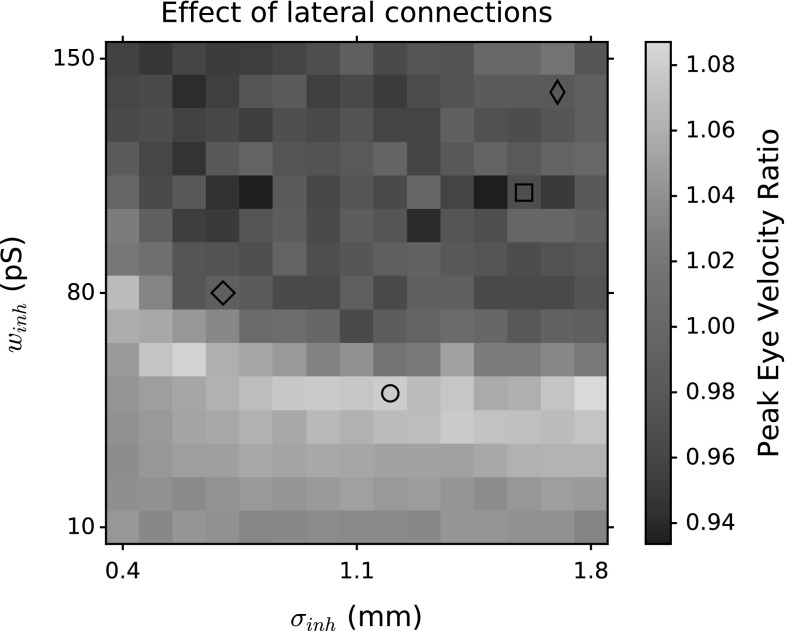
Ratio of peak eye velocity with lateral connections to the peak eye velocity without lateral connections, for different lateral inhibition parameters (inhibitory width, $\sigma _{\mathrm{inh}}$, and inhibitory strength, $w_{\mathrm{inh}}$) and fixed excitatory lateral connections: $w_{\mathrm{exc}}=160$ pS, and $\sigma _{\mathrm{exc}}=0.4$ mm. All peak eye velocities are computed for a 21$^{\circ }$ saccade amplitude (see Methods 2.9). Four parameter sets are marked by different symbols (see Fig. 14). Note that we used the parameter set $\circ$ throughout the paper to demonstrate the network activities. This parameter set was given by the genetic algorithm

In this one-dimensional network model, we quantified the effect of lateral connections on the network performance by the resulting changes in peak eye velocity. The ratio of peak eye velocities for the network with and without the selected lateral connections are shown in Fig. 13 for different lateral interaction schemes. Single neurons’ firing frequencies increase as the lateral excitation increases. This results in higher spike counts and higher peak firing rates overall. Yet, since the linear ensemble coding scaling factor, $\kappa$, is calculated by the population spike count, eye kinematics depend on temporal activity of the population. Synchronized bursts result in higher peak eye velocities. Therefore, Fig. 13 reads that low $w_{\mathrm{inh}}$ values result in faster saccades compared to the baseline eye movement generated by the network activity when the lateral interactions are omitted. The $w_{\mathrm{inh}}$ and $\sigma _{\mathrm{inh}}$ pairs that generate the fastest eye movements lie around around $w_{\mathrm{inh}}=50-70$ pS (yellow band). For higher inhibitory strengths, the peak eye velocity may even become slower than the baseline. That is not because of a lack of synchrony of the neurons, but because of stretched firing profiles. As the inhibition builds up too fast, the bursts are no longer gamma-shaped.

**Fig. 14:**
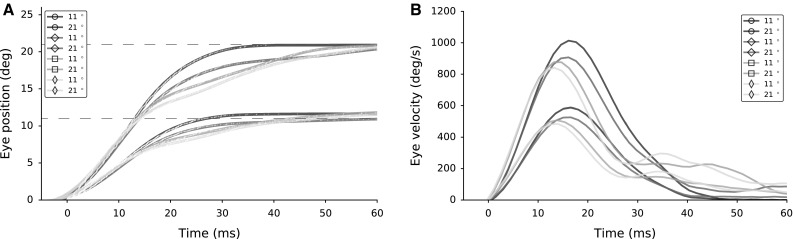
**a** Eye movements to two targets (at 11$^{\circ }$ and 21$^{\circ }$) for the four different lateral interaction parameter sets marked in Fig. 13 are shown in shades of green and blue, respectively. **b** The associated velocity profiles show markedly different kinematics. Not all lateral interaction profiles lead to optimal saccade behavior (only the two darkest curves correspond to optimal saccades; $\circ$ in Fig. 13)

To illustrate the behavioral differences, saccades to targets at 11$^{\circ }$ and 21$^{\circ }$ are generated from the population activities of four parameter sets (marked $\square$, $\Diamond$, $\circ$ and $\diamond$) in Fig. 14. Figure 14a shows the eye displacements for all four parameter sets to saccade targets. $\circ$ results in the highest peak eye velocity and more normometric eye displacements for both targets. Associated eye velocity profiles in Fig. 14b illustrates that high inhibition ($\Diamond$ and $\square$) results in unusually slow eye movements with a long tail.

## Discussion

In the present study, we studied the properties of a simple, one-dimensional spiking neural network model that accounts for the measured activity patterns of cells in the motor SC and embeds the spatiotemporal transformation that underlie fast saccadic eye movements. In short, the total ongoing spike count of the recruited population in the motor map encodes the saccade trajectory (spatial code), whereas the instantaneous firing rates of the recruited cells are responsible for optimizing the saccade velocity profile (temporal code).

**Mechanism** Our model describes a biologically plausible scheme, which suggests that the observed burst profiles of SC cells may result from distinct biophysical properties of the neurons, in combination with lateral excitatory–inhibitory interactions within the motor map. In our model, the SC activity is not suppressed by any type of external inhibition, as the SC cells only receive a translation-invariant excitatory input burst from an upstream (cortical) source. After the initiation of spiking activity by the distributed cortical input, the intrinsic neural adaptation of the SC neurons, together with the lateral inhibition, builds up and terminates the neural activity. Adequate tuning of the parameters of the SC cells ensured a fixed number of spikes in the bursts of cells located near the center of the recruited population across the motor map, and to burst durations and firing rates that systematically varies with the neuron’s location in the map (Goossens and Van Opstal 2006, 2012).

We varied the adaptation time constant in a linear way as function of the rostral-to-caudal map coordinates from 80 ms to 10 ms, and the top-down projections from the upstream input source varied parabolically from approximately 16–6 nS (Fig. 6). We constrained the parameter pairs to result in different burst profiles that elicited the same number of 20 spikes per saccade (the average number of spikes reported by Goossens and Van Opstal 2006). This fixed spike count results from the neural adaptation mechanism that is incorporated in the AdEx neuron model. The adaptation current, *q*, acts as an intrinsic inhibitory current on the membrane potential, *V*, to prevent repetitive high-frequency firing under constant current stimulation. The temporal evolution of the adaptation current affects the response profile of the neuron to tonic input. In this way, neural adaptation can offer a basis for varying the ISI distributions (burst profiles) of SC neurons. We targeted the adaptation time constant, $\tau _q$, as a critical tuning parameter because it determines how fast the adaptation current will decay. Since *q* acts on the membrane potential as an inhibitory current, $\tau _q$ affects the instantaneous change in the membrane potential, *V*, indirectly, and consequently the burst profile of the neuron. Furthermore, varying the adaptive properties also affects the neurons’ susceptibility to synaptic input. As a result, the spike counts decrease for larger $\tau _q$ values in Fig. 4a because the accumulated total adaptation current, *q*, competes with the total synaptic input to the neuron. As a result, the driving conductances should also vary among the SC neurons to ensure a fixed number of spikes throughout the motor map (i). Figure 4b depicts the burst profiles of neurons with the same adaptation time constants as in Fig. 4a for different synaptic strengths, $w_{i}^\mathrm {F-S}$. In that way, the neurons can generate gamma function-like saccade-related bursts with the observed properties (i, iv).

The SC firing patterns all result from intrinsic properties of SC neurons, rather than from external inhibitory suppression, or from negative feedback. Most previous models of the saccadic system assumed that the main-sequence kinematics of saccades results from a nonlinear local feedback mechanism in the reticular formation that is known to embed the saccadic burst generators (for example (Robinson 1975; Jürgens et al. 1981; Scudder 1988, reviewed by Girard and Berthoz 2005). These models are all based on the assumptions that (1) the SC only encodes the desired eye displacement vector and (2) the saccade kinematics are fully determined downstream in the brainstem. The models proposed by Arai and Keller (2005), Trappenberg et al. (2001) and Marino et al. (2011) are the most recent and prominent ones, all following that notion (introduced by Robinson 1975). In addition, these collicular models account for trajectory variations that result from competing visual stimuli (Arai and Keller 2005) or to saccade reaction-time differences when there are parallel inputs to the SC (Trappenberg et al. 2001; Marino et al. 2011). These latter models aimed to account for saccade reaction times through the rise in neural activity prior to the saccade onset and did not focus on a collicular role underlying the saccade kinematics (which in our model is determined by the instantaneous burst activity during the saccade). Moreover, none of these previous models explore neural computations at the spiking level, although they all incorporated lateral interactions within SC.

**Table 2:** Overview of the properties of SC activity and the underlying intrinsic SC mechanisms

Aspect of SC activity	Model Mechanism
Burst profiles (gamma-bursts) (Fig. 8a)	Translation-invariant input activity temporal profile (Fig. 3) through FEF-SC projections, $w_n^{\mathrm{F-S}}$, and adaptive current, *q*, acting on the membrane potential
Fixed number of spikes of central cells’ bursts (i, Fig. 8b)	Interplay between adaptation time constant, $\tau _q$, and synaptic input strengths from the FEF-SC projections, $w_n^{\mathrm{F-S}}$ (Fig. 4)
Gradient of peak firing rates of central cells (iv, Fig. 8b)	Location-dependent variation of adaptation time constant, $\tau _q$, and synaptic input strengths from the FEF-SC projections, $w_n^{\mathrm{F-S}}$ (Fig. 6)
Synchronization of bursts in population (iii, Fig. 9)	Soft WTA lateral interactions in motor map (Fig. 7)
Fixed number of spikes of total population (active gaussian populations, Fig. 10a)	Translation-invariant input, a fixed density of SC neurons, and the mechanism that creates the movement field of the SC cells (Fig. 11a)
Saccade-dependent temporal activities of the gaussian populations (Fig. 10b)	Topographic distinct properties (Fig. 6) and lateral interactions (Fig. 7)
Spike count of a given SC neuron for different saccade amplitudes (ii, movement fields, Fig. 11a)	Log-polar relationship of the afferent mapping (Eq. 3) and the neuron’s spike count in active gaussian populations (Fig. 10)
Saccade-dependent burst profiles of a given SC neuron (Fig. 11b)	Soft WTA lateral interactions in motor map (Fig. 7)
Saccadic motor commands (Fig. 12a, b)	Dynamic linear summation of spike vectors (Eqs. 1–2)

Recently Morén et al. (2013) proposed a spiking neural network model of the SC to explore the generation of saccadic command signals. That model is considerably more complex than our minimalistic one-layer model, as it consists of seven interconnected SC layers of cells, all with different synaptic properties and neurotransmitter systems. Yet, despite its complexity, the Moren model does not capture the essential feature of spike synchronization in their population of recruited cells. It is not clear either, given the complexity of their model system, how to successfully include this property into their model.

In our spiking neural network model, the total burden of nonlinear saccade kinematics is embedded at the level of SC motor map, while the brainstem circuitry may act as a simple linear feedback system (Fig. 1). Hence, the firing rates of the SC neurons directly reflect the saccade velocity profiles. Note, however, that according to the linear ensemble coding scheme, the spiking profiles of individual cells in the recruited population do not necessarily need to correlate well with the instantaneous eye velocity profile. Even when all bursts would have a rectangular shape (synchronized and scaled according to their movement fields), the total population would still reflect the saccade velocity profile quite well, despite the fact that none of the individual cells would correlate at all with eye velocity. However, the fact that the individual cell activities do resemble the velocity profile is a strong indication that they indeed encode eye velocity.

We excluded feedback from the brainstem saccade generator as a putative mechanism to stop the SC bursts, because perturbation experiments have shown that SC activity does not encode dynamic eye motor error (Goossens and Van Opstal 2000a, b; Soetedjo et al. 2002; Kato et al. 2006; Munoz et al. 1996). On the other hand, there is physiological evidence that saccade-related SC neurons have distinct intrinsic membrane properties (Grantyn et al. 1983) and that the bursting profiles might be associated with NMDA receptor activation (Saito and Isa 2003; Isa and Hall 2009). For our network model, we hypothesized that the rostral-to-caudal gradient of peak firing rates results from location-dependent biophysical properties of the SC neurons, which is a major novel aspect, in relation to the earlier proposed models. We had also hypothesized, however, that such a mechanism would not be sufficient to generate the synchronized population activity. Indeed, although a more rostral and caudal cell at the same distance from the central neuron both receive the same excitatory spike trains from the FEF, they will fire differently (i.e., at different peak firing rates and different burst durations) because of their different biophysical properties (illustrated in Fig. 4). Furthermore, since the total input to a neuron depends on its distance from the center, the time it takes for a neuron to reach its bursting regime gets slightly longer too, leading to increased desynchronization of the neural activity.

It is therefore not to be expected that the parameters, which determine the intrinsic biophysical properties of the AdEx neurons, can somehow be tuned to lead to better synchronization, as these parameters primarily influence the peak firing rates and the number of spikes in the bursts (Fig. 6). As a result, these neurons will never be able to account for population synchronization without lateral interactions, because also the burst shape should vary prominently with the saccade in which the cell participates, rather than by its mere location within the motor map. This paper demonstrates that lateral excitatory–inhibitory interactions can provide for a mechanism to make this happen. However, it cannot be excluded that the population synchrony may be caused by some upstream excitatory–inhibitory mechanism, e.g., the involvement of FEF neurons (providing the local excitation) in combination with the substantia nigra, which could provide the global inhibition (e.g., Hikosaka and Wurtz 1985). However, so far, there is no evidence that FEF neurons, for example, display any relation to the kinematic encoding of saccades as observed in the SC.

On the other hand, the presence of lateral interactions within the SC motor map (Meredith and Ramoa 1998; Munoz and Istvan 1998) is well established. Recent in vitro multichannel local field potential studies have suggested Mexican hat-type lateral interactions in the intermediate and superficial layers of the SC (Phongphanphanee et al. 2011, 2014). Those intrinsic circuit properties do not require the motor SC to take part within a feedback loop to generate the observed systematic firing characteristics. Indeed, in our model the saccades are driven in a feedforward way by the SC population. An overview of the underlying intrinsic mechanisms that result in the required SC properties (i–iv) is given in Table 2.

It should be noted that it is possible that other parameter settings, and/or connectivity schemes, could potentially produce a similar behavior of the SC cells. Figures 9 and 13 highlight that the parameters of the Mexican hat profile significantly affect the synchrony in the population activity, as well as the ensuing saccade kinematics. Our analysis also shows that a range of parameters (yellow band in Fig. 13) can produce the appropriate behavior through synchronized population activity. Although beyond the scope of this study, we expect that other lateral interaction profiles, differing from the ideal Gaussian, but with appropriately weighted excitation and inhibition strengths, may yield similar results.

**Optimal controller** The decay of peak firing rates along the rostral–caudal axis in the motor map has recently been argued to embed the nonlinear main-sequence properties of the saccade kinematics (saturating peak eye velocity; Van Opstal and Goossens 2008). As the function of saccades is to bring the fovea *as fast and as accurately as possible* to the peripheral target of interest, the main sequence may at first glance seem to counteract this requirement. In early models of the saccadic system, the main sequence properties were typically assigned to (passive) saturation of brainstem burst neurons, which reach peak firing rates up to 1000 spikes/s for large saccades and hence clearly reach neural saturation levels. Indeed, plotting the instantaneous peak firing rate of a brainstem burst neuron against the instantaneous motor error results in a unique phase curve that resembles the amplitude–peak eye velocity relation of saccades (Van Gisbergen et al. 1981). Because of this, the input–output relation of brainstem burst neurons has been modeled by the same nonlinear, saturating curve. In this way, these models “explain” the saccade main sequence by assuming a nonlinearity in the brainstem pulse generator. However, we recently highlighted several problems with this interpretation (Goossens and Van Opstal 2012): first, the input signal to the burst neurons is not known, as single-cell recordings can only reveal their output. Therefore, whether the input signal represents dynamic motor error, or a desired eye velocity signal, remains speculative at best. Second, brainstem burst neurons are not the only ones to fire at such extreme firing rates during saccades, as also oculomotor neurons (OMNs) and medial vestibular neurons easily reach these levels. Nonetheless, in the earlier models the OMNs are considered to be linear. Hence, placing the saturating nonlinearity only at the pulse-generating neurons may be somewhat arbitrary. Third, even when a given neuron may have a saturating input–output characteristic, the total neural population may still act as a linear controller. Taken together, the need for a nonlinear transformation at the level of the brainstem burst generator is questionable. To support this argument, we demonstrated that a linear brainstem model, driven by the measured unfiltered spike patterns of recorded SC neurons can indeed fully account for the main-sequence properties of saccades (Eq. 1; Goossens and Van Opstal 2006).

We therefore argued that the recruited population in the SC motor map acts as a nonlinear vectorial pulse generator, which provides scaled (and coupled) horizontal and vertical eye velocity signals to the brainstem pulse generators. As a result, the SC population automatically encodes a straight, shortest path, saccade trajectory to the target, which would be expected for a system that needs to be as fast as possible. One may wonder why saccades have to obey a saturating main sequence, especially when neural saturation in the brainstem is not needed to account for the saccade kinematics. Theoretical studies (Harris and Wolpert 1998, 2006; Tanaka et al. 2006; Van Beers 2008) have shown that the main sequence might in fact result from an optimal control strategy for a system that has to cope with speed–accuracy trade-off in the presence of peripheral uncertainty of the visual field (low spatial resolution of the retina) together with signal-dependent noise in the neural commands. Therefore, the spatial gradient in the peak firing rates of SC neurons may reveal a deliberate *design* within the system in order to *ensure* a saturating, but optimal, kinematic main sequence. In support of this theory, we observed several other properties of the SC firing rates, which are incorporated in our model.

Through our proposed winner-take-all lateral connectivity scheme, the central cell imposes its own temporal profile on all cells in the population. This secondary mechanism thus leads to two important properties of SC burst behavior, which were so far not accounted for by other SC spiking models (Morén et al. 2013): (i) a large degree of burst synchronization of the cells in the recruited population, and (ii) the burst profile of a particular SC cell is not determined by its location in the motor map, but by the saccade for which it is recruited (Goossens and Van Opstal 2012). Both properties further support the notion that the SC motor map functions as an optimal controller for saccades: burst synchronization leads to a maximally powerful impulsive input to the brainstem burst generator, which thus ensures an optimal acceleration of the eye (see Fig. 12). Indeed, the acceleration phase of saccades is virtually independent of the saccade amplitude, with a nearly fixed duration of about 15–20 ms. The latter is presumably mainly determined by the (unavoidable) dynamics of the oculomotor plant (i.e., the short time constant of the eye muscles). Our model accounts for these optimal kinematics through lateral interactions in the SC motor map (Figs. 12, 13, 14).

**Current limitations** As a proof of principle, we restricted our model to SC activity for visually evoked saccades in one dimension. The hypothesized input from FEF drives the SC motor map by a translation-invariant input pattern (Schlag-Rey et al. 1992; Segraves and Park 1993) that signals only the location of the saccade target, while providing the same temporal pattern for all saccade amplitudes. Segraves and Park (1993) showed that the FEF activity starts well before the saccade onset and continues for about 90 ms after the saccade is executed. In this model, we have only assumed that the SC is activated by the same input pattern for any saccade amplitude. Clearly, to explain the emergence of different firing patterns of SC cells, despite the same FEF input, the parameters of the SC cells had to vary in a systematic way. Although this simple scheme can explain a wide variety of phenomena with a minimum number of assumptions, several important issues are not yet incorporated in our model:

1. The model needs to be extended to two dimensions to generate saccades in all directions. The current model architecture, however, allows for a relatively straightforward extension and parameter tuning to a two dimensional network.
2. Electrical microstimulation in the SC with a train of brief high-frequency pulses elicits normometric saccades with normal kinematics, although the stimulation train has no relationship to either saccade duration, or saccade velocity (Robinson 1972; Van Opstal et al. 1990; Stanford et al. 1996). This may seem problematic for a population model that precisely encodes the saccade metrics and kinematics by its detailed firing patterns. Models that assume that the SC does not play any role in encoding the saccade kinematics regard the temporal firing profiles of SC neurons as immaterial, as only the *location* of the population matters in driving the saccade. If true, our dynamic ensemble coding model is in big trouble. Although one may assume that the population activity of SC cells would mimic the rectangular, fixed-frequency envelope of the stimulation train, there is actually no evidence that this is indeed the case. It should be realized that the activity patterns resulting from microstimulation are not known. As the electric field from the microstimulating electrode rapidly decays with distance, it is conceivable that microstimulation only activates a few neurons near the electrode tip and that the population activity is the result of intrinsic network synaptic transmission. A recent study in FEF has indeed suggested that stimulation at intensities of 10 microamps excites only a few neurons near the electrode (Histed et al. 2009). Katnani and Gandhi (2012) studied the effect of SC microstimulation frequency and intensity on the saccadic behavior and showed that different microstimulation procedures result in the same behavior, provided that the total injected charge is equivalent. These results support the idea that once a small set of neurons gets activated, they build up a population activity that yields a normal saccade. Although our model can in principle capture the transmission of neural activity from a centrally activated cell to the rest of the population through the lateral excitatory–inhibitory connectivity scheme, we have not yet incorporated such a mechanism to its full extent.
3. Our experiments have demonstrated that SC activity during blink-perturbed saccades has a transient decrease in the overall firing rates throughout the entire SC. However, the elicited number of spikes for the (goal-directed) saccade remained unaffected (Goossens and Van Opstal 2000b), although the saccades lasted much longer, were highly variable, and had much lower peak velocities. Currently, our model has a relatively strong dependence on the input current. In an improved version of the model, the SC population activation should rely less on the details of the input current and set up its population activity mainly through lateral connections and intracollicular dynamics (see also the previous point). The external input may therefore act predominantly as a trigger for this process.
4. A more complete model will have to include the separate controls of the eye and head motor systems as well, in combination with the vestibular system, to generate gaze shifts with varying contributions of eyes and head, and concomitant changes in the gaze kinematics. Our recent recordings indicate that changes in initial eye position in the orbit strongly influences the gaze shift kinematics. Interestingly, this factor also modulates the SC firing rates (in line with their expected role in kinematics control), as well as a subtle concomitant change in the number of spikes (unpublished observations).
